# Recognition and Management of Serotonin Toxidrome in the Emergency Department—Case Based Review

**DOI:** 10.3390/jpm12122069

**Published:** 2022-12-15

**Authors:** Bianca Codrina Morarasu, Adorata Elena Coman, Cristina Bologa, Catalina Lionte, Ovidiu Rusalim Petris, Alexandr Ceasovschih, Victorita Sorodoc, Raluca Ecaterina Haliga, Gabriela Puha, Alexandra Stoica, Oana Sirbu, Mihai Constantin, Laurentiu Sorodoc

**Affiliations:** 1 2nd Internal Medicine Clinic, ‘‘Sfântul Spiridon’’ Emergency Clinical County Hospital, 700111 Iasi, Romania; 2 Preventive Medicine and Interdisciplinary Team Department, “Grigore T. Popa” University of Medicine and Pharmacy, 700150 Iasi, Romania; 3 Internal Medicine Department, “Grigore T. Popa” University of Medicine and Pharmacy, 700111 Iasi, Romania; 4 Nursing Department, “Grigore T. Popa” University of Medicine and Pharmacy, 700111 Iasi, Romania

**Keywords:** serotonin syndrome, olanzapine, overdose

## Abstract

Serotonin syndrome (SS) is a clinical toxidrome with high variability in clinical practice. It develops due to increased serotonin levels in the central nervous system. With an underestimated frequency, SS can develop following an overdose, a therapeutic dose increase, or drug to drug interaction of at least one serotonergic agent. It can present with autonomic signs, neuromuscular changes and an altered mental status. However, history and clinical examination are key features to formulate the diagnosis. Treatment options consist of supportive measures, discontinuation of the offending agent and certain therapeutic agents previously reported to improve outcomes. Physicians have limited experience with SS, partially due to the lack of its identification in clinical practice. Therefore, we have integrated, in a narrative review, the case of a young male with SS following an atypical antipsychotic overdose superimposed on chronic treatment with agents previously known to produce SS.

## 1. Introduction

Serotonin syndrome (SS) is the result of increased serotonin levels in the central nervous system [1]. It can progress to a life-threatening condition, secondary to intentional self-poisoning, drug to drug interaction or therapeutic medication use. Multiple drugs have been correlated with the development of SS, however, selective serotonin reuptake inhibitors (SSRIs), serotonin norepinephrine reuptake inhibitors (SNRIs) and monoaminoxidase inhibitors (MAOIs) are the most common cause [2]. Cases of SS secondary to atypical antipsychotic agents have been described [3], but it remains a controversial topic. We present the case of an Olanzapine induced SS, integrated into a narrative literature review.

## 2. Case Report

A 21-year-old male patient was brought in by ambulance after he was found by the family in an unconscious state, lying on the floor. He had taken an overdose of Olanzapine 5 mg (7 tablets) and Lorazepam 1 mg (2 tablets) from the chronic medication of another family member. The patient’s past medical history was significant for severe depression with previous suicidal attempts; the patient had been under chronic treatment with Trazodone, Aripiprazole and Sodium valproate for the past two years. The social history was remarkable for gambling dependence without the associated use of illicit drugs. In the emergency department (ED), the patient presented altered consciousness, agitation, and a Glasgow coma score (GCS) of 8, raised heart rate (120 beats per minute) and blood pressure (160/80 mmHg), tachypnoea (respiratory rate 22 per minute), and normal temperature. Clinical examination showed bullous fixed drug eruptions (similar to ’’barb burns’’ lesions) in the right cheek, chin, both knees, anterior right thigh and 3rd, 4th and 5th finger of the left hand, and 5th finger of the right hand. The neurological examination was remarkable for spontaneous lower limb bilateral clonus. A limited cranial nerve examination showed no significant abnormalities. The pupils were equal bilaterally, round, of around 5 mm, and reactive to light. The tonus was increased with lower limb hyperreflexia. There were no peripheral focal neurological findings, and meningeal maneuvers were negative. Head computer tomography (CT) ruled out an organic cause, a chest X-ray showed no pneumonia, an abdominal ultrasound was normal, and an electrocardiogram (ECG) showed sinus tachycardia. Blood tests performed in the emergency department were unremarkable, except for leukocytosis (WCC 18,000/m^3^) and raised arterial lactate (37 mg/dL). A urinary toxicology screen was negative. The patient was subsequently assessed by the Intensive Care Unit (ICU) team, who decided to intubate and mechanically ventilate the patient due to a persistent altered consciousness and inability to maintain the airways. He was transferred to our Toxicology ICU, where, after 24 h, he developed pyrexia (40.2 degrees Celsius), persistent tachycardia and hypertension (190/80 mmHg). The patient underwent supportive treatment with sedation and intravenous fluid hydration, with a good clinical response within the next 36 h. After 3 days in the ICU, he was transferred to our Toxicology Department, observed for the next 24 h and further discharged with a follow-up by the psychiatry service.

## 3. Epidemiology of Serotonin Syndrome

The incidence of SS is generally thought to be underestimated, probably due to the high variability in clinical presentation and lack of identification by the treating physician. A large number of general physicians (85.4%) were unaware of SS when questioned via a questionnaire for a study conducted in 1999 [4]. 

The literature has described SS in different ages, from new-borns to the elderly population, with a mean age of 30 to 40 years in the adult population [5]. In recent years, the use of serotonergic agents has increased, leading to a higher incidence of this condition. During 2015–2018, Centers for Disease Control and Prevention (CDC) reported that 13.2% individuals aged over 18 were taking antidepressant agents, with a higher use among women (17.7%) compared to men (8.4%) [6]. In Europe, in 2008, there was an average number of 2.3 to 6.4 SSRI prescriptions per patient, with approximately 936 users of antidepressants per 10,000 person-years [7]. Another Swedish study showed a number of 1,215,558 prescriptions for SSRI between 2005 and 2013 [8]. Rates of MAOI use have declined over the years, with less than 0.1% of individuals receiving prescriptions in 2005 in the United States [9], and patients receiving MAOI treatment in the United Kingdom falling from 9000 to 4000 during 2004–2013 [10].

Between 2002 and 2016, 54,410 individuals in the United States were exposed to an overdose of SSRIs, with 102 deaths, representing the 9th most frequent cause of mortality by drug overdose [11]. An estimated incidence of SS is 0.5 to 0.9 cases/1000 patient/months of treatment with SSRI monotherapy, as reported by the British National Health Service [4], with a mortality rate of 14–16% in people taking and overdosing on SSRI [12]. Another large retrospective cohort study included 15 million patients taking serotonergic agents, with data extracted from the Veterans Health Administration and commercially insured patients records, showed an incidence of SS between 0.07 to 0.19% [13]. Interestingly, SS may arise in infants born from mothers taking serotonin agents. It presents with a wide variety of symptoms when taken in the third trimester, such low Apgar score, low birth weight and respiratory distress, as well as excessive sleepiness, poor feeding and weight loss when taken during breastfeeding. This might constitute a topic that can be addressed in future studies [14].

## 4. Pathophysiology

Serotonin (5-hydroxytryptamine, 5-HT) is a monoamine neurotransmitter synthetized from the amino acid tryptophan. The metabolic pathway uses multiple mechanisms, feed-back loops, and enzymes. Tryptophan hydroxylase 1 and 2, are essential for the synthesis of serotonin in the gastrointestinal (GI) tract and the central nervous system (CNS), respectively. Serotonin is stored in the presynaptic vesicles and released into the synaptic cleft, following axonal depolarization [15]. Through a receptor dependent activity, in the CNS, serotonin modulates high cognitive functions, including mood, anger, aggression, attention, memory, or appetite, while in the GI tract it regulates motility and intestinal secretion or pancreatic enzymes activity [16]. It is also involved in platelet activation and coagulation cascade [17] in cardiac function [18], in pulmonary vessels [19], in the hypothalamic–pituitary–adrenal axis, glucose metabolism [20], and in the genitourinary function [21]. There are seven classes of serotonin receptors that mediate the serotonin effects and the associated features of serotonin syndrome. 5-HT_1_ receptors are mediated by adenylyl cyclase and represent the main target for anti-migraine drugs, such as sumatriptan/ zomitriptan as they have a high density in the limbic system. 5-HT_2_ receptors stimulate phosphoinositide-specific phospholipase, and their antagonism leads to the therapeutic effect of antipsychotic drugs such as risperidone. The 5-HT_3_ receptor is a ligand-gated cation channel predominantly expressed by neurons, and represents the pharmacological site for anesthetic and antiemetic agents. The 5-HT_4_ family influences gastric motility, while the 5-HT_5_ family is less known. 5-HT_6_ and 5-HT_7_ receptors are still under investigation since many antipsychotics and antidepressants act through antagonism [22,23]. 

Increased levels of serotonin in the CNS represent the foundation for SS development [24]. SS is the result of serotonin over-stimulation through drug agonism, antagonism or a combination of both, which may develop after an overdose of this medication. It may also occur after a therapeutic dose is started, increased, or when serotonergic drugs are associated with similar agents that alter the serotonin metabolism [25,26]. Out of all serotonin receptors, 5-HT_1A_ and 5-HT_2A_ are the most often involved in SS. Genetic susceptibility may be a culprit for SS development and variability, especially in the context of therapeutic doses [27]. Single nucleotide polymorphism in the 5-HT_2A_ receptor subtype and homozygous carriers of the C102 allele of the same receptor are considered to provide a higher risk of serotonin induced adverse drug reactions [28,29,30]. Polymorphism of the CYPs in the serotonin metabolism, causing altered drug pharmacokinetics and low metabolizing activity, has been described in several case reports [31,32]. Additionally, different levels of receptor sensibility may contribute. 5-HT_1A_ receptors have higher serotonin affinity, which is associated with milder presentation, while 5-HT_2A_ activation has been associated with life threatening SS. Other factors may influence the pharmacology of serotonergic drug, such as age, sex, nutrition, health status [33] or hormonal balance. Estrogen increases serotonin concentrations through tryptophan hydroxylase and prolongs its action through the antagonism of the serotonin reuptake transporter [34]. The role of this interaction has already been characterized in a variety of conditions and needs further investigations in regard to SS.

Multiple pathophysiological pathways are probable in our case. The use of serotonin receptor agonists, namely 5-HT_1A_, and serotonin reuptake inhibitors as chronic treatment led to increased levels of serotonin the CNS. The superimposed overdose with an atypical antipsychotic, with an affinity for 5-HT_2A_/_2C_, 5-HT_3_ and 5-HT_6_ receptors, escalated serotonin concentrations and precipitated the clinical syndrome. Other factors, such as the patient’s poor general health status and nutritional deficiencies, might have contributed.

## 5. Drugs Associated with SS

A variety of drug classes have been associated with SS, such as antidepressants, monoaminoxidase inhibitors, antiemetics, antimigraine agents, illicit drugs, over the counter drugs or herbal supplements [35]. They can be divided into four large classes depending on their action: serotonin precursors, inhibitors of serotonin metabolism, inhibitors of serotonin reuptake, and molecules that sensitize the serotonin receptors or have a direct action (Table 1). The addition of a drug acting through cytochrome P450 inhibition can result in accumulation of serotonergic agents [33].

An association of MAOIs and SSRIs or SRNIs can cause life-threatening SS [36]. Other concerning combinations include MAOIs and triptans, antibiotics (particularly Linezolid) and meperidine. It seems that an association of antidepressants and opiates represented the most frequent cause (16.1%) for SS, followed by drug overdose (15.4%) in a metanalysis, described by Werneke et al. [37].

Atypical antipsychotics (AA) are a relatively new class of medication acting on a wide range of receptors. They can be divided into two main categories depending on their capacity for dopamine receptor antagonism. AA have been increasingly used in a variety of conditions such as schizophrenia, depression, bipolar disorder, or agitation in geriatric patients [38]. Olanzapine is an AA with an affinity for 5-HT_2A_/_2C_, 5-HT_3_, 5-HT_6_ receptors, antihistamine H_1_ receptors, dopamine D_1_, D_3_, D_4_, D_5_ receptors, as well as cholinergic muscarinic receptors. Studies have demonstrated a higher affinity for 5-HT_2_ receptors compared to dopaminergic D_2_ receptors. It undergoes first-pass metabolism through cytochrome P450 2D6 with different pharmacokinetic properties and two-phase elimination when taken in overdose [39]. Case reports of olanzapine-induced SS have been published, sometimes as a single agent [40], but more often when used in combination with other agents such as mirtazapine [41], clomipramine [42], metoclopramide [43] or duloxetine [44]. It is important to note our patient’s chronic medication. Aripiprazole is an atypical antipsychotic with antidepressant properties with a partial D_2_ and 5-HT_1A_ agonist and antagonist of serotonin 5-HT_2A_ receptors. It demonstrated high affinity for dopamine D_2_, D_3_, serotonin 5-HT_1A_, 5-HT_2A_, histamine H_1_ and alpha-1 adrenergic receptors when studied in vitro. Aripiprazole has been associated with serotonin syndrome [45]. In the context of 5-HT_2A_ antagonism, there is enhanced 5-HT_A1_ agonism, which increases serotonin levels, potentiates the effects of other drugs, and increases receptor sensitivity to serotonin; this explains the development of SS [40]. Trazodone is an antidepressant, triazolopyridine derivative which inhibits serotonin uptake. It has been associated with SS when associated with other agents [46]. Valproic acid and its sodium salt (sodium valproate) are antiepileptic drugs with an effect on serotonin receptors. It leads to increased levels of serotonin in the central nervous system, providing an anticonvulsant effect. This mechanism may constitute the reason for the development of SS in patients taking valproic acid, especially in association with other potent agents [47].

## 6. Clinical Signs and Symptoms

The triad of autonomic signs, altered mental status, and neuromuscular changes characterizes SS [48]. Neuromuscular changes are generally the most prominent. Clonus is one of the most important clinical features. It can be induced in mild cases, becomes spontaneous in severe SS, but may disappear in life-threatening toxicity [49]. Other associated signs are tremor, akathisia, hyperreflexia, and muscular rigidity. Eye examination can show mydriasis and different types of nystagmus, more frequently binocular horizontal pendular nystagmus [1,50]. Initially, these symptoms may only involve the lower limbs, then may become generalized, and in severe cases mask the other symptoms. An altered mental status can fluctuate from excitement or agitation, to confusion, seizures, and coma. Autonomic signs are characterized by diaphoresis, hyperthermia, tachycardia, hypertension, tachypnoea, vomiting and diarrhea. Hyperthermia, with a temperature exceeding 41 degrees Celsius, should be considered a feature of severe SS. In a metanalysis by Prakash et al. [51], fever was reported in 61% of patients and tachycardia in 36%; these were the most common findings in the studied population. 

SS is diagnosed based on clinical presentation. A thorough examination of the patient’s medical history, looking for chronic medication for depression or long-term pain, use of illicit drugs, such as ecstasy, amphetamine, or cocaine, and over the counter medication such as St John’s wort, appetite suppressants or tryptophan, should be performed. There should be careful consideration of whether a recent increase in dose, addition of a second drug, or resumption of an agent with prolonged half-life has been performed in a patient’s chronic medication [15]. The onset of symptoms ranges between minutes to weeks, and their resolution may be within 12 to 24 h after drug withdrawal and supportive care. Clinical examination represents the subsequent key diagnostic step. In certain cases, a rapid progression to severe SS can occur and a thorough assessment may not be possible [52,53]. Patients may require ICU treatment due to lactic acidosis, rhabdomyolysis, seizures, coma, liver or renal failure [54,55] and, in this context, pose diagnostic difficulties. A mild case can be easily outlooked as the patient can present with anxiety, mild tremor, or diaphoresis. In this context, when a serotonergic agent is initiated, resumed or a dose adjusted, clinicians should be aware of possible SS development and closely monitor the patient. The latter, as well as the family, should be informed about possible symptoms and alert the physicians to the early stages of SS. Tachycardia, hypertension, and a core temperature higher than 40 degrees Celsius are common features for moderate SS. The patient associates hyperreflexia with clonus on patellar tendon reflex examination, repetitive behavior, pressured speech, and mild agitation. 

In our case, the medical history provided by the family characterized a patient with a history of depression and previous suicidal attempts, for which he was taking three antidepressants previously reported to determine SS. We do not have a clear timeline about the onset of symptoms as he was found lying on the floor with empty blisters in his hand. It should be noted that another family member was taking treatment with Olanzapine. The patient exhibited clonus, one of the most important signs associated with SS. Altered mental status, together with hyperthermia, tachycardia and hypertension, led us to consider SS as the main diagnosis. 

There is no specific laboratory test to diagnose SS. Only in severe cases, metabolic acidosis, rhabdomyolysis, increased levels of liver enzymes, kidney function tests or coagulation tests abnormalities can be determined. Imaging, such as head CT, lumbar puncture, and ECG, should be performed to exclude other organic causes [56]. We performed a full set of bloods (full blood count, renal, liver function, electrolytes, inflammatory markers, urine toxicology) and imaging tests (chest X-ray, ECG, head CT) to exclude potential organic causes. Compared to other toxidromes [57], the variability of SS and the limited number of cases does not permit a standardized battery of tests to diagnose or to predict an outcome in SS. However, different authors have suggested a constellation of specific signs and symptoms to increase the sensitivity and specificity of SS diagnosis. Sternbach [58], Radomski [59] and Hunter [60] are the three diagnostic classification systems used worldwide. Sternbach characterized the SS criteria in 1999, based on the observation of 38 psychiatric patients, followed by Radomski’s review in 2001 (62 cases). With far more patients (2222), in 2003, Dunkley redefined a part of the diagnostic criteria, focusing on neuromuscular symptoms and excluding myoclonus. Currently, the Hunter criteria are the gold standard, as the author proved higher sensitivity (84% vs. 75%) and specificity (97% vs. 96%) compared to Sternbach’s criteria [60]. The latter classification system lacks inclusion of mild cases as it only considers moderate to severe symptoms, and includes symptoms that may be determined by other drug or cerebellar signs which are not specific to SS [1]. Despite this, it remains one of the important tools in identifying SS. Next, Radomski added rigidity to Sternbach’s clinical features of SS. The Hunter criteria focused on neuromuscular symptoms, introducing clonus, and removing myoclonus. It consists of a decision tree and is characterized by ingestion of a serotonergic agent plus the following physical findings: spontaneous clonus, inducible clonus associated with agitation or diaphoresis, inducible clonus or ocular clonus associated with hypertonia and hyperthermia, ocular clonus associated with agitation or diaphoresis, tremor, and hyperreflexia [59]. Our patient took an atypical antipsychotic, which has been previously reported to determine SS. He also met the Hunter criteria with spontaneous clonus, agitation, and hyperreflexia. In contrast to the aforementioned findings, a systematic review of 412 cases of SS were reanalyzed by applying the Hunter, Sternbach, and Radomski criteria. The former had the worst performance, as it would have missed 37% cases of SS, while Sternbach and Radomski would have missed 8% and 11%, respectively [61].

## 7. Differential Diagnosis

Differential diagnosis should be made with other toxin-induced hyperthermic syndromes. Neuroleptic malignant syndrome (NMS), anticholinergic/antimuscarinic syndrome, and malignant hyperthermia are the main conditions to consider. A broad differential diagnosis should also be considered for conditions such as sepsis, febrile neurologic illness (such as autoimmune encephalitis), nonconvulsive status epilepticus, hyperthyroidism, and rhabdomyolysis [62], or stiff person syndrome [63].

SS should primarily be differentiated from NMS. The latter is a toxidrome induced by dopamine antagonists characterized by hyperthermia, rigidity, altered mental status and autonomic dysfunction [64]. The two syndromes share multiple common clinical features, and some are of the opinion that they may represent different stages of the same condition. In both cases, patients will present with hyperthermia, altered mental status, autonomic dysfunction, and muscle rigidity. Hyperthermia can be present in around 88% of patients with NMS. The main differentiating features are the time of onset and evolution of symptoms. NMS develops within 7 to 10 days, and it may persist for weeks after discontinuation of the offending agent. The motor findings are characterized by bradykinesia and diffuse rigidity (lead-pipe or cogwheel) in the absence of clonus and hyperreflexia, which is characteristic to SS [65]. The main differential diagnosis in our case was with NMS. This was considered because the drugs that our patient had taken have an added dopaminergic antagonistic action. The presence of an altered mental status posed diagnostic difficulties, firstly, due to inability to take history and, secondly, due to the wide variety of conditions presenting with this feature. Altered mental status, hyperthermia and autonomic dysfunction resembled characteristics for both SS and NMS in our patient. However, the drug history, and most importantly, spontaneous clonus, guided us towards SS. 

Anticholinergic toxicity is characterized by central and peripheral features. It is frequently encountered in the ED as there are over 600 identified compounds with anticholinergic action. The syndrome can present with altered mental status, such as anxiety, agitation, coma, or seizures, secondary to a reduced cholinergic activity in the CNS. The peripheral findings provide features distinguished from SS: normal reflexes, dry oral mucosa, erythematous skin, and urinary retention [66]. 

Malignant hyperthermia is a life-threatening pharmacogenomic condition affecting skeletal muscle. It arises in susceptible individuals after the exposure to volatile anesthetic drugs, e.g., halothane, isoflurane, or succinylcholine [28]. The genetic predisposition has been linked to mutations within the ryanodine receptor subtype 1 and dihydropyridine receptor, as they are essential regulators of cytoplasmic calcium. The clinical picture is characterized by an unexplained increase in end-tidal carbon dioxide, with subsequent tachypnoea, tachycardia, hyperthermia, and muscle rigidity. In contrast to SS, patients exhibit hyporeflexia, mottled skin and mortis-like rigidity. 

Hyperactive delirium shares multiple common findings with SS. This is particularly important in elderly patients where specific drug agents causing SS are used, but delirium is frequently encountered [21]. Altered mental status, with agitation, tremor, tachycardia, hypertension, diaphoresis can arise in both conditions. Myoclonus, hyperreflexia, and hyperthermia should prompt SS as a possible diagnosis.

The sudden discontinuation of antidepressants may have features of SS. Symptoms consist of malaise, nausea, changes in sleep, mood, or appetite, but the duration is less than a week, with spontaneous resolution [67]. 

## 8. Treatment

The mainstay treatment consists of supportive measures and the discontinuation of the offending agent. Where indicated, the patient should be admitted to a high dependency unit and close vital sign monitoring should be ensured [24]. If possible, the patient should be kept in a quiet and calm environment. Symptoms should start to improve within 24 h. 

Mild cases can benefit from fluid replacement and sedation where necessary. Benzodiazepines are indicated in cases of agitation, regardless of the severity of SS. Diazepam or lorazepam improve survival due to the added benefit of lowering blood pressure, heart rate and temperature, by controlling the hyperadrenergic state [36]. If persistent, severe tachycardia and hypertension can be treated with short-acting esmolol or nitroprusside. Long-acting betablockers should be avoided due to the undesirable effect of masked tachycardia, hypotension, and possible shock. In case of hypotension (MAOI interaction), inotropic support with norepinephrine or epinephrine can be used [1,33]. Dopamine should generally be avoided due to a possible exaggerated hemodynamic response. 

Moderate cases should be admitted for cardiac monitoring and may require the addition of a serotonin antagonist if there is no response to benzodiazepines [68]. Butyrophenones, such as haloperidol, should not be used, due to hyperthermia possibly worsening through the anticholinergic mechanism. 

Chlorpromazine and cyproheptadine have been extensively used. Both agents have 5-HT_2A_ action, but cyproheptadine has an additional 5-HT_1A_ receptor antagonist mechanism. It may be more effective than chlorpromazine as it has been shown to prevent life-threatening hyperthermia, provide symptomatic relief, and even complete symptom resolution in some cases [69]. The initial dose of cyproheptadine is 12 mg with a 2 mg increase every 2 h, if needed, with a total of 32 mg over 24 h if symptoms do not improve. The maintenance dose is 8 mg every 6 h. It may cause sedation as a side effect. Chlorpromazine is currently less recommended due to adverse effects. It can cause orthostatic hypotension, worsening hyperthermia and dystonia [70,71].

Severe or life-threatening cases should be admitted to the ICU where orotracheal intubation, active cooling, management of autonomic instability and the use of Cyproheptadine might be required. Hyperthermia control is critical as it is often responsible for the severity of serotonin syndrome [51]. It develops as a result of increased muscle activity, as opposed to a central hypothalamic temperature set point. All measures should aim to minimize muscle activity with external cooling methods, and the avoidance of physical restraint due to enhanced isometric muscle contraction with subsequent severe lactic acidosis. When temperature exceeds 41.1 degrees Celsius, the patient should be intubated. Vecuronium can be used for muscle paralysis, as opposed to succinylcholine that may cause hyperkalemia and worsening rhabdomyolysis. Antipyretic agents play no role [24]. Although other numerous agents have been used to treat SS, there is no specific antidote therapy. Alternative therapeutic resources, such as dantrolene, bromocriptine, propranolol or chlorpromazine, have been previously used, but they are not currently recommended [72].

## 9. Conclusions

SS remains a challenging condition due its wide variety of symptoms, lack of objective standardized diagnostic tests and limited research in the literature. When facing such an emergency, the most important step is to carefully evaluate the patient’s drug history, present complaints, and perform a detailed clinical examination. The combination of multiple agents targeting serotonin receptors is a common strategy for treatment-resistant depression. This strategy, however, creates the perfect environment for the potential development of SS, especially in the context of an overdose. In our case, the patient was taking chronic treatment with strong serotoninergic agents, and the overdose of Olanzapine led to a serotonin receptor blockage, increased serotonin levels and the development of SS. The clinical judgement becomes crucial when there is limited available data, or the patient has severe consciousness impairment. Clinicians should therefore bear in mind that SS can be considered as a differential diagnosis for a broad range of conditions and more important when there is relevant drug history. Moreover, prior to initiating a medication acting on serotonin receptors, careful assessment and monitoring should be conducted. 

## Figures and Tables

**Table 1:** Classes of drugs and mechanism of action associated with SS.

Drug Class	Serotonin Precusors	Inhibitors of Serotonin Metabolism	Inhibitors of Serotonin Reuptake	Serotonin Receptor Sensitisers/Agonists
**SSRIs/SNRIs**			Citalopram, escitalopram, fluoxetine, fluvoxamine, paroxetine, sertraline, duloxetine, venlafaxine, milnacipran	
**Trycyclic Antidepressants**			Amitriptyline, nortriptyline, trimipramine, clomipramine, imipramine, amoxapine	
**MAOIs**		Linezolid, tedizolid, selegine, rasagiline, tranylcypromine, isocarboxide, phenelezine, moclobemide		
**Antidepressants**			Bupropion, trazodone, nefazodone	Mirtazipine, olanzapine, trazodone
**Opiates**			Methadone, tramadol, fentanyl, tapentadol, pethidine, meperidine	Fentanyl, meperidine
**Antiepileptics**			Valproate, carbamazepine	
**Antiemetics**			Ondansetron, granisetron, metoclopramide	Metoclopramide
**Illicit drugs**	Cocaine		Cocaine, MDMA, cathiones	Buspirone, LSD
**Others**	Tryptophan	Methylene blue, Procarbazine, St John’s Wort, Triptans	St John’s Wort	Triptans, lithium
